# Nightmares in Patients with Major Depressive Disorder, Bipolar Disorder, and Psychotic Disorders: A Systematic Review

**DOI:** 10.3390/jcm9123990

**Published:** 2020-12-09

**Authors:** Marine Ambar Akkaoui, Michel Lejoyeux, Marie-Pia d’Ortho, Pierre A. Geoffroy

**Affiliations:** 1 Department of Psychiatry and Addictive Medicine, Assistance Publique-Hôpitaux de Paris (AP-HP), University Hospital Bichat, 46 rue Henri Huchard, 75018 Paris, France; michel.lejoyeux@aphp.fr (M.L.); pierre.a.geoffroy@gmail.com (P.A.G.); 2 Centre Psychiatrique d’Orientation et d’Accueil (CPOA), GHU Paris—Psychiatry & Neurosciences, 1 rue Cabanis, 75014 Paris, France; 3 Etablissement Publique de Santé Mentale de Ville Evrard, 202 Avenue Jean Jaurès Neuilly-Sur-Marne, 93300 Neuilly-sur-Marne, France; 4 Université de Paris, 75018 Paris, France; 5 Université de Paris, NeuroDiderot, Inserm UMR1141, F-75019 Paris, France; marie-pia.dortho@aphp.fr; 6 Department of Physiology and Sleep Center, Assistance Publique-Hôpitaux de Paris (AP-HP), University Hospital Bichat, 46 rue Henri Huchard, 75018 Paris, France

**Keywords:** nightmare, bad dreams, sleep disorders, mood disorders, psychotic disorders, suicide

## Abstract

Chronic nightmares are very common in psychiatric disorders, affecting up to 70% of patients with personality or post-traumatic stress disorders. In other psychiatric disorders, the relationships with nightmares are poorly known. This review aimed to clarify the relationship between nightmares and both mood and psychotic disorders. We performed a systematic literature search using the PubMed, Cochrane Library and PsycINFO databases until December 2019, to identify studies of patients suffering from either a mood disorder or a psychotic disorder associated with nightmares. From the 1145 articles screened, 24 were retained, including 9 studies with patients with mood disorders, 11 studies with patients with psychotic disorders and 4 studies with either psychotic or mood disorders. Nightmares were more frequent in individuals with mood or psychotic disorders than in healthy controls (more than two-fold). Patients with frequent nightmares had higher suicidality scores and had more frequently a history of suicide attempt. The distress associated with nightmares, rather than the frequency of nightmares, was associated with the severity of the psychiatric disorder. Further studies assessing whether nightmare treatment not only improves patient–sleep perception but also improves underlying psychiatric diseases are needed. In conclusion, nightmares are overrepresented in mood and psychotic disorders, with the frequency associated with suicidal behaviors and the distress associated with the psychiatric disorder severity. These findings emphasize major clinical and therapeutic implications.

## 1. Introduction

Sleep disorders are often implicated in the clinical course of psychiatric disorders. Among these sleep disorders, insomnia and nightmares are very common in clinical practice associated with psychiatric disorders [1]. Nightmares are associated with increased psychological distress [1], worse physical health outcomes [2], and increased risk of self-harm and suicide [3,4]. Whereas episodic nightmares are very common with a prevalence of about 35–45% (one nightmare per month) [5,6,7], the prevalence of chronic nightmares is relatively low in the general population, ranging from 2–8% [5,6,7,8,9,10]. Nevertheless, the prevalence of these chronic nightmares seems significantly more frequent in individuals with psychiatric disorders [11]. Indeed, chronic nightmares are very common in psychiatric disorders, affecting up to 70% of patients with personality or post-traumatic stress disorders [2,12,13]. In other psychiatric disorders such as psychotic and mood disorders, which are very frequent, the relationships between nightmares and these disorders is poorly known. The prevalence of nightmares is consistent across several countries and cultures, including the United States, Canada, Europe, Japan, and Middle East [14]. The International Classification of Sleep Disorders, third edition (ICSD-3) defines nightmares as “coherent dream sequences that seem real and become increasingly more disturbing as they unfold. Emotions usually involve anxiety, fear or terror. The dream content most often focuses on imminent physical danger to the individual, but may also involve other distressing themes” [15,16]. Nightmares occur frequently in the context of REM sleep, and would usually awaken the sleeper, with a recollection of disturbing mental activity [10].

Episodic nightmares should be distinguished from a nightmare disorder. The fifth edition of the Diagnostic and Statistical Manual of Mental Disorders (DSM-5) [17] defines nightmare disorder according to five diagnostic criteria: (A) repeated occurrences of extended, extremely dysphoric and well-remembered dreams that generally occur during the second half of the major sleep episode; (B) the individual rapidly becomes oriented and alert on awakening from the dysphoric dreams; (C) the sleep disturbance causes clinically significant distress or impairment in social, occupational, or other important areas of functioning; (D) the nightmare symptoms are not attributable to the physiological effects of a substance; (E) coexisting mental and medical disorders do not explain the predominant complaint of dysphoric dreams. The DSM-5 specifies the nightmare disorder’s duration: acute (<1 month), sub-acute (more than 1 month but less than 6 months) or persistent (>6 months). The DSM-5 also specifies the severity of the disorder: severe (one episode per night) and moderate disorder (one or more episode per week).

Sleep complaints directly impact mental health and predict suicide attempts independently of all psychopathologies and sociodemographic characteristics [18]. More specifically, nightmares are overrepresented in almost all psychiatric disorders and are also associated with suicidal thoughts and behaviors, as well as suicide, independently of psychiatric disorders and symptoms [19,20,21]. Moreover, nightmares are also associated with other suicide risk factors, including hopelessness and depression [22,23] which predict suicidal ideation and behavior [24,25]. Nightmares seem to be more frequent in patients with major depressive disorders (MDD), bipolar disorders (BD), and schizophrenia than in the general population [26]. It has also been proposed that nightmares and psychotic symptoms represent a common domain with shared pathophysiology [27]. Nevertheless, some findings are controversial, including whether these associations correlate or not with the intensity of psychiatric symptoms, or if the frequency and/or the intensity of nightmares are associated with psychiatric disorders and suicidal behaviors. In addition, the question of whether nightmares are a specific or unspecific symptom shared by all or some psychiatric disorders still needs to be unraveled. Finally, although nightmares were associated with an increased risk of suicide in the general population, we wanted to clarify whether this association also exist in individuals with psychiatric disorders.

In this context and the absence of extensive reviews on mood and psychotic disorders, this systematic review aimed to clarify the association between nightmares and these psychiatric disorders by analyzing all studies assessing nightmares and mood disorders (major depressive disorders and bipolar disorders), and psychotic disorders (schizophrenic and non-schizophrenic disorders). The objective was to specify the prevalence of nightmares in patients with mood disorders or psychotic disorders, and their association with psychiatric manifestations and suicidal risk.

## 2. Methods

We followed the Preferred Reporting Items for Systematic Reviews and Meta-analyses (PRISMA) [28] guidelines for the literature search and analysis.

### 2.1. Eligibility Criteria

We decided to include only studies examining mood or psychotic disorders because of their high prevalence and the absence of systematic reviews.

We included all studies meeting the following inclusion criteria: (A) must be an original paper (including case report, case study and case series, and epidemiological studies), (B) must have enrolled patients with a primary diagnosis of either: mood disorder (bipolar disorder or major depressive episode), psychotic disorder (schizophrenic and non-schizophrenic disorders), suicide, according to DSM or International Classification of Disease (ICD) criteria [17,29], and assessed by standardized scales, (C) must have studied nightmares, with any type of nightmare assessment, and (D) must study adolescents or adults over 18 years of age.

Studies were excluded if: (A) they enrolled patients with another primary psychiatric disorder (personality disorders, anxiety disorder, phobia, Post-Traumatic Stress Disorder (PTSD), eating disorder, attention-deficit hyperactivity disorder (ADHD), addictions, autism spectrum disorders) or non-psychiatric disorders (dementia, cognitive disorders), (B) they studied other parasomnias, (C) they were not in English or French, (D) patients were younger than 18 years old or older than 80 years old, (E) they were a systematic review or a meta-analysis, (F) nightmares were induced by treatments, and (G) participants were selected from the general population.

### 2.2. Search Strategy

We searched on PubMed, Cochrane Library and PsycINFO and attempted to screen all the scientific literature until December 2019. The following terms were used for the literature search: (“nightmare” (All Fields) OR “dream” (All Fields) OR “parasomnia” (All Fields) OR “nightmare” (Medical Subject Headings (MeSH)Terms)) AND (“psychosis” (All Fields) OR “psychosis “(MeSH Terms) OR “hallucination” (All Fields) OR “schizophrenia” (All Fields) OR “bipolar disorder” (All Fields) OR “bipolar disorder” (MeSH Terms) OR “suicide” (All Fields) OR “suicide” (MeSH Terms) OR “depression” (All Fields) OR “major depressive episode” (All Fields) OR “depressive disorder” (All Fields) OR “depressive disorder” (MeSH Terms) OR “manic” (All Fields)). Relevant studies were identified and their reference lists were hand searched.

### 2.3. Study Selection

**Figure 1:**
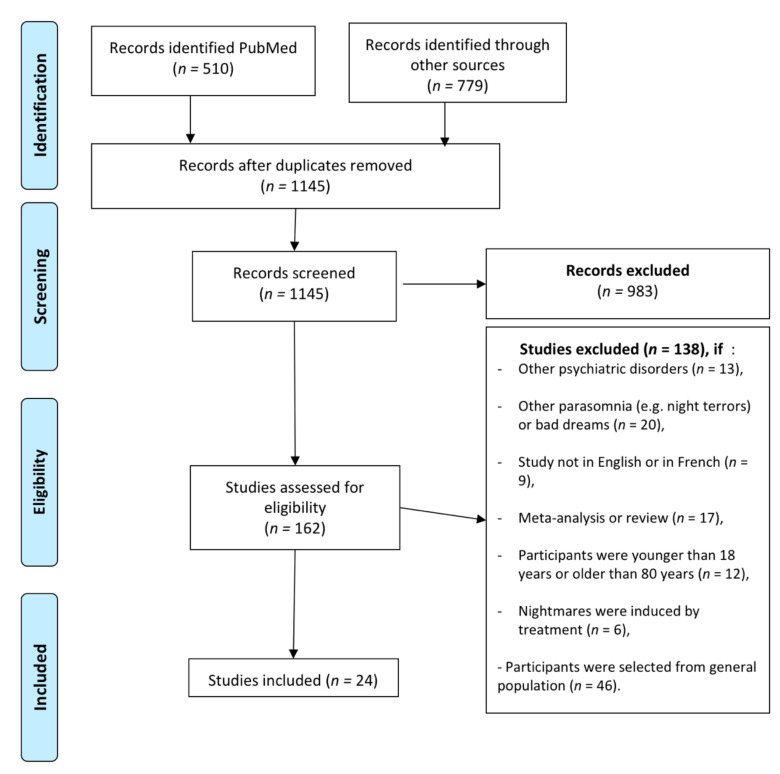
Flow diagram of nightmares and psychiatric disorders (mood and psychotic disorders) selection process.

Two authors (M.A.A. and P.A.G.) independently screened for the titles of potentially eligible publications. Some papers were excluded at this stage (See Figure 1). After review of abstracts and papers, likely inclusions were assessed by MAA and PAG who independently extracted all information on age, sex, main diagnosis, nightmares frequency and distress, other comorbid sleep disorders and psychiatric disorders including post-traumatic stress symptoms and suicidality.

### 2.4. Frequent-Nightmare Definition

As no consensus exists regarding frequent-nightmare definition, this definition varies considerably between studies. In the table, the frequency definitions used in reported studies are indicated as follow: * Frequent nightmares were defined as ≥2/week, **: ≥1/week, ***: monthly to weekly, **** no definitions.

## 3. Results

Among the 1145 screened articles, 24 were included in this systematic review (Figure 1). The diagnostic criteria used in the retained studies, as well as the characteristics of studies that were included, are summarized in Table 1, Table 2 and Table 3.

The results were separated into four categories: prevalence, association with symptoms, association with suicide, and treatment.

### 3.1. Nightmares and Mood Disorders

We found nine studies on nightmares and mood disorders (Table 1).

#### 3.1.1. Major Depressive Disorders (MDD)

##### Nightmare Frequency in MDD

Few studies have estimated the prevalence of nightmares in patients suffering from MDD. One study found that patients with MDD experienced nightmares more than twofold than healthy controls (mean of 44.6 nightmares per year in patients with MDD, versus 18 nightmares per year in healthy controls; the prevalence of nightmares was 16.7% and 4.9%, respectively) [25].

##### Suicidality and Nightmare in MDD

Patients with MDD and nightmares seem to have a higher risk of suicidality than MDD patients without nightmares. Agargun et al. found a significantly higher suicidality scores in patients with MDD and nightmares than without nightmares [29]. They also found, in a subgroup analysis, that this difference was significant in women, but not in men. In another study, Agargun et al. [32] found that nightmares were more frequent in MDD patients with suicidal attempts than in those without suicidal attempts (86% vs. 71% respectively). Marinova et al. explored the suicide risk in patients with unipolar recurrent depression and confirmed that patients with nightmares had a significantly higher suicidal score than those without nightmares (2.36 vs. 1.00 on the Hamilton Depression Rating Scale (HDRS) subscale for suicide) and had significantly more frequently a history of suicide attempt (35% vs. 6%) [22].

##### Nightmares and MDD Symptoms

Patients were more likely to experience frequent nightmares (≤2/week) when they had a MDD with melancholic features than without nightmares (respectively 90% of nightmares vs. 56%) [31]. Moreover, in the same study, nightmares were significantly more frequent in depressed patients with terminal insomnia (e.g., “early morning waking”) than without insomnia, regardless of melancholic features (90.1% vs. 19.4% respectively) [31].

Agargun et al. [32] studied 100 patients with melancholic features, and observed an increased frequency of nightmares and of middle and terminal insomnia in patients who committed a suicide attempt than those without suicide attempt (nightmares, *n* = 27 e.g., 96% in suicide attempt group, vs. *n* = 57 e.g., 79% in the non-suicide attempt group). The authors found no significant differences in non-melancholic depressed patients for suicide attempt, both for nightmares and insomnia. Moreover, nightmares were more common in major depressed patients with melancholic features (84%) than without melancholic features (57%).

##### Treatment of Nightmares in MDD

Thünker et al. [33] tested the effectiveness of standardized nightmare therapy based on image rehearsal therapy (IRT), in MDD patients suffering from nightmares, and showed a decrease in the nightmare frequency. Woo et al. [35] found an improvement for nightmares in MDD patients after four sessions of eye movement desensitization and reprocessing (EMDR). Li et al. [34] found that MDD patients with nightmares and insomnia had fewer chances to be in remission at four years of MDD (29.8% of remission in patients with nightmares vs. 47.3% in patients without nightmares). Moreover, they found that residual nightmares, but not residual insomnia, were significantly associated with suicidal ideation in patients remitted from major depressive disorders.

#### 3.1.2. Bi-Polar Disorders

Marinova et al. [22] tested the hypothesis that nightmares were associated with an elevated suicidal risk in depressed patient, differentiating bipolar depression from recurrent depression. They found a significantly higher frequency of nightmares in unipolar- than in bipolar-depression (64% vs. 25%). There were no differences in the bipolar depression group for the suicide risk in patients with nightmares, compared with those without nightmares. However, the sample of depressed patients with bipolar disorder was small (*n* = 8 patients), which may explain the absence of significant results despite the large differences of frequencies reported. Lai et al. [36] also found that frequent nightmares (not defined) in bipolar or unipolar depression were associated with a higher risk of suicidal ideation (OR = 2.88) and attempts (OR = 1.89), after adjustment for age and sex.

Of notice, the scientific literature is very poor regarding nightmares and mania. Only one study [30] reported nightmares in three patients suffering from a first manic episode. The three patients reported at least one prodromal nightmare before their first episode [30].

### 3.2. Nightmares and Psychotic Disorders

We found 11 studies on nightmares and psychotic disorders (Table 2).

#### 3.2.1. Nightmare Frequency in Psychotic Disorders

Nightmares are the second most frequent sleep disorder in patients with psychotic disorders, after insomnia, with a prevalence of frequent nightmares (defined as ≤1/week) ranging from 9.0% to 55% [45,46]. Insomnia and nightmares were frequently comorbid in patients with psychotic disorders. Accordingly, patients who had frequent nightmares (defined a ≤1/week) were more likely to report frequent insomnia (37.1% vs. 17.6%). No data examined whether the severity of insomnia symptoms correlates or not with nightmare frequency or intensity.

#### 3.2.2. Nightmares and Psychotic Symptoms

Fennig et al. [38] reported the case of a 78 year old patient, which saw a transition from a nightmare to a brief psychotic episode. The delirium had the same thematic as his recurrent nightmare of the last three years. He was treated with neuroleptics, with no recurrences of nightmare after six months of follow up. Another case report [39] found an association between decompensation and nightmares in a 40 year old woman suffering from schizophrenia: she had insomnia and nightmares the week before relapse, with the same thematic of delusion and hallucination as in her nightmares.

Three studies found that patients with schizophrenia reported nightmares significantly more frequently than healthy controls [26,41,42]. Moreover, patients with schizophrenia had a higher score of distress related to nightmares than healthy controls [41]. Neither significant correlations between nightmare frequency and intensity of psychotic symptoms measured by the Positive And Negative Syndrome Scale (PANNS), nor correlations for depressive symptoms (Beck scale for depression) and nightmare frequency were found in those patients with schizophrenia [41]. However, nightmare distress, rather than nightmare frequency, was correlated with delusional severity, depression, anxiety, and stress [43].

One study found in exploratory measures in fourteen patients with at-risk mental states for psychosis (ARMS) that they experienced significantly more nightmares compared to healthy controls [41], even ARMS patients who did not receive any treatment.

#### 3.2.3. Suicidality and Nightmares in Psychotic Disorders

In one study [45], nightmare frequency was associated with a lifetime history of suicide attempts but did not predict an increased risk of suicide attempt over the eight years follow up period. In the same study, insomnia and nightmare were significantly associated with an increased risk of suicide attempts (lifetime history and during the follow-up period) [45].

#### 3.2.4. Treatment of Nightmares in Psychotic Disorders

One case report [37] was of a 38 year old patient with schizophrenia. The patient had daily nightmares about the devil in the shape of a snake. The patient was treated by desensitization of the fear of snakes, with good efficacy on nightmares. This treatment efficacy was maintained over time, and the patient did not experience any nightmares during the two years follow-up [37].

Sheaves and al. [42] experienced imagery rehearsal therapy (IRT)—a cognitive-behavioral treatment for reducing the frequency and intensity of nightmares—for the treatment of nightmares in the context of psychosis in six patients, and observed an improvement on vividness and intensity of nightmares, without a decrease in nightmare frequency. One other non-randomized study found efficacy of IRT on the emotional response of nightmares in psychotic patients [50]. Interestingly, one pilot randomized clinical trial has evaluated IRT to reduce nightmare severity in 24 patients with persecutory delusions, with large size effect improvements in nightmare severity and insomnia [47]. In addition to IRT, the intervention included cognitive behavioral therapy techniques to target identified causal factors for nightmares. Large effect size improvements in nightmare severity and insomnia were reported post-treatment (4-weeks) and maintained at follow-up (8-weeks) [47].

### 3.3. Study Comparing MDD and Schizophrenic Patients

We found four additional studies comparing different psychiatric disorders regarding nightmares (Table 3).

One study [25] compared nightmare frequency in MDD and schizophrenic patients with a population without psychiatric disorders and found that nightmares were significantly more frequent in psychiatric patients, but did not find any differences between MDD and schizophrenic patients (respectively 17% and 4.9% in patients with psychiatric disorders and healthy controls).

In one cohort study [48] with 165 patients (33.3% MDD, 6.6% psychotic disorder, 24.8% alcohol/substance misuse disorder, and 16.3% anxiety disorder), frequent nightmares (not defined) were more present in patients with a higher score of suicidality irrespective of underlying psychiatric disorder. Another study [19] conducted in the same cohort of patients found that patients with more than one suicide attempt had more frequent nightmares than those with one unique suicide attempt. Moreover, after a two-month follow-up, patients with more than one suicide attempt were more likely to have persistent nightmares during the follow-up period (46% vs. 14% respectively).

More recently, Lamis et al. [49], in a cohort of 172 patients with bipolar disorders (29.7%), MDD (11.6%), and psychotic disorders (46.6%), found that patients who reported nightmares (defined as weekly to monthly), compared to patient who reported yearly or no nightmares, were younger and more likely to have been hospitalized for a recent suicide attempt, with no differences between these psychiatric disorders.

## 4. Discussion

Nightmares were more frequent in individuals with mood disorders and psychotic disorders [26,42] than in individuals without psychiatric disorders. This is concordant with the observed higher prevalence of sleep complaints in psychiatric patients than in the general population [51]. Table 4 summarizes key findings from this systematic review of nightmares in mood and psychotic disorders (Table 4).

Nightmares were found to be associated with higher suicidality in several studies, including suicidal ideations and attempts, in patients with mood disorders [23,31,32,35,36], or psychotic disorders [45]. In the general population, nightmares are also associated with a higher risk of suicidal thoughts, suicide attempts, or death by suicide [19,20,21]. Moreover, several studies found frequent co-occurrence of insomnia and nightmares. Insomnia is one of the most common comorbid sleep disorders associated with psychiatric disorders [51,52], and is by itself associated with a higher risk of suicidal ideation in healthy and psychiatric populations [53]. Whereas insomnia is much more often screened for by psychiatrists, being a core symptom in the classification of some psychiatric disorders such as MDD or Bipolar disorder [16], the identification of nightmares is rare and is not included in mood or psychotic disorders classification. In this context, practitioners frequently consider nightmares and disturbing dreams as secondary symptoms, with no predicting or therapeutic relevance. However, even if most of the studies assessing nightmares and suicide are cohorts or case series, and that controlled studies are needed to clarify the role of nightmares on suicidal behavior, the present review suggests that patients should be systematically screened for recurrent or frequent nightmares, as they are both very frequent and seem to be associated with a higher risk of suicide [15,34,44,45,54].

The correlation between nightmares and intensity of symptoms in mood disorders or psychotic disorders is not clear. Indeed, rather than nightmare frequency, nightmare distress may be more specifically associated with psychotic and depressive symptoms [42]. No studies have reported relationships between nightmare distress and depressive symptoms in patients with MDD, nor the relationship between nightmare distress and suicidality in patients with MDD or psychotic disorders. In the general population, nightmares have been associated with hallucinatory experiences [55] and with psychotic-like experiences [46,47,48,49,50,51,52,53,54,55,56].

We decided to exclude all studies with patients under 18 years old because nightmares are more common in children and teenagers, and to avoid potential confusion factors. Nevertheless, one longitudinal study [57] interestingly found that nightmares at 12 years old were a significant predictor of psychotic experiences at 18 years old, after adjustment for possible confounders. This report is in line with our observations previously mentioned. Moreover, Michels et al. found that ARMS patients had more frequent nightmares than healthy controls [41], suggesting that nightmares may be present at a very early stage of the disease.

The exact pathophysiology of nightmares in patients with mood and psychotic disorders is not entirely known. In a recent review, Gieselman et al. hypothesized the etiology of nightmares by hyperarousal and impaired fear extinction, with facilitating factors such as traumatic experiences and childhood adversity, trait susceptibility, maladaptive cognitive factors, and physiological factors [58]. Levin et al. proposed that nightmares reflect problems with the fear extinction function of dreaming [10]. Schredl et al. propose that certain nightmare themes, such as suicide, are of particular interest because they may be related to the psychopathology of waking life [59]. Further studies are expected to better unravel these physio-pathologies and specificities in psychiatric disorders, since nightmares in the context of trauma, stress, delirium, anxiety, or depressed mood may have different pathways and causes.

### 4.1. Clinical and Therapeutic Implications

Suicide, which is the first cause of death among young people, is associated with several modifiable or non-modifiable risk factors, so it is important to be able to identify and manage [17]. Nightmares have been identified as one of the modifiable risk factors for suicide, with specific treatments, such as Image Rehearsal Therapy or Systematic Desensitization and Progressive Deep Muscle Relaxation training for treatment of idiopathic nightmares, or Prazosin if nightmares are associated with of Posttraumatic Stress Disorder (PTSD) [58,60]. Furthermore, as mentioned above, nightmares may be associated with early stages of psychotic or mood disorders, and its treatment may prevent the conversion to a full psychiatric disorder.

### 4.2. Limitations

This work clearly emphasizes a need to use standardized definitions of nightmares across studies, as we observed a lack of consensus criterion. Indeed, “frequent nightmare” was differently defined from one study to another, and sometimes not defined at all. With our code with asterisks (from * to **** when no definitions were proposed, see our methods), we tried to clarify this issue. We plead for standardized use of either the DSM-5 definition or systematic use of published questionnaires such as the Mannheim Dream Questionnaire (MADRE) [60]. DSM-5 defines a mild nightmare disorder as less than one episode per week on average, a moderate disorder as one or more episodes per week, but less than nightly, and a severe disorder as nightly episodes. An acute episode has a duration of 1 month or less, a sub-acute episode a duration between 1 to 6 months, and chronic nightmares endure for 6 months or longer (APA, 2013). Instruments to assess nightmare frequency and nightmare distress exist such as the Nightmare Frequency Questionnaire (NFQ) [61] or the MADRE [60]. The use of such instruments should be more generalized.

Some caveats and limitations of the existing scientific literature reviewed here should be emphasized. First, most studies of nightmares and mood disorders [62] assessed major depressive disorders (MDD), without any information about the unipolar of bipolar subtype of depression. This could bias results; for example, Marinova et al. [22] found a difference in the frequency of nightmares between unipolar and bipolar depression. Patients with unipolar depression and nightmares were more likely to have suicidal thoughts than those without nightmares; this difference was not found in patients with bipolar disorder (although this was a smaller group with underpowered statistics). Second, patients with PTSD or recent trauma were either not screened, nor always excluded from the studies. Yet nightmares are one of the mains symptoms of PTSD and stress-related disorders. This may have been a confounding factors in reported studies [46,48]. Third, there was no information of comorbid personality disorders; as in PTSD it may have been a confounding factor, as it is known that some personality disorders such as borderline personality are more associated with nightmares [2]. Fourth, most cohort studies examined the frequency of nightmares in their patients and looked for an association with suicidality. A significant majority did not report information on the severity of psychiatric symptoms. Patients who had more frequent nightmares were more likely to have suicidal thoughts or attempts but may also have had a higher intensity in their symptoms, which may have led to higher suicidality. Fifth, there was little information about the distinction between bad dreams/nightmares in reported studies. Nightmares are different from bad dreams since nightmares awaken the sleeper [10]. Sixth, only few studies mentioned their patient medication. However, nightmares have been found as being a side effect of some antipsychotics and antidepressant treatments, and so may have been a confounding factor as well [63]. Finally, all the studies were based on clinical evaluation (self-report nightmares or clinician interview), which can have led to a memory bias. There were no laboratory examinations of nightmares with more objective measures (except in the study of Lusignan et al. [40], who explored dreams and not nightmares).

## 5. Conclusions

Nightmares are much more frequent in patients with mood and psychotic disorders than in the general population. Patients with nightmares, compared to those without nightmares, also suffered from more suicidal thoughts and attempts. Little information was available on the association of nightmares and the severity of psychiatric symptoms, and this relationship warrants further investigations. Besides, rather than the frequency of nightmares, it may be the distress associated with nightmares that is correlated with the severity of the psychiatric disorder. Importantly, nightmares seem to respond to specific therapies such as IRT, and further investigations are needed to see if they should be used as add-on treatments and may prevent conversion to full psychiatric disorders or poorer outcomes of existing mood and psychotic disorders. Further studies assessing whether nightmare treatment not only improves patient–sleep perception but also underlying psychiatric diseases are definitively needed.

Legends: The literature search was performed from PubMed, Cochrane Library and PsycINFO electronic database, using the following terms: (“nightmare” (All Fields) OR “dream” (All Fields) OR “parasomnia” (All Fields) OR “nightmare” (MeSH Terms)) AND (“psychosis” (All Fields) OR “psychosis “(MeSH Terms) OR “hallucination” (All Fields) OR “schizophrenia” (All Fields) OR “bipolar disorder” (All Fields) OR “bipolar disorder” (MeSH Terms) OR “suicide” (All Fields) OR “suicide” (MeSH Terms) OR “depression” (All Fields) OR “major depressive episode” (All Fields) OR “depressive disorder” (All Fields) OR “depressive disorder” (MeSH Terms) OR “manic” (All Fields)).

## Figures and Tables

**Table 1:** Mood disorders and nightmares.

Authors Years	Study Design	Aims	*n*	Age (Years)	Sex F	Diagnosis Criteria for Psychiatric Disorder	Nightmares: Diagnosis and Severity	Other Evaluation	Main Results	Comments
Agargu, 1998 [29]	Controlled study	To examine the association between repetitive and frightening dreams and suicidal tendency in patients with major depression	MDD, *n* = 29	31.6	69% (0)	DSM-III-R criteria for major depression	All patients received an oral definition of nightmare. Nightmare frequency Frequent nightmare if ≥2/week	SADS suicide subscale ≥ 3 BDI	Mean suicidality score was higher in MDD patients with nightmares than in the control group: 4.2 vs. 2.9 in the second group, *p* = 0.014. 72% of patients with nightmares classified as being suicidal vs. 47% in the non-nightmare group.	Controlled group: depressed patients with dreams but no nightmares (*n* = 31)
Agargun, 2003 [30]	Cases reports	To discuss relationship between dreams and the first episode of the illness in term of mood regulatory functions of dream	ME, *n* = 3	29.0	33.3% (*n* = 3)	DSM-IV criteria	-	-	All patients had one nightmare before decompensation of their first manic episode	
Besiroglu, 2004 [31]	Controlled study	To investigate the relationship between nightmares ant terminal insomnia un unipolar depressed patients with melancholic features	MDD, *n*= 82 (unipolar)	32.4	58.5% (*n* = 48)	DSM-IV criteria	All patients received an oral definition of nightmare. Nightmare frequency Frequent nightmare if ≥ 2/week	HDRS (items 6, 7 and 8 for insomnia)	Nightmares were more frequent in the melancholic feature group (90%, *n* = 74), than in non-melancholic group (56%, *n* = 42), *p* < 0.001. Nightmares were significantly more frequent in the patients with terminal insomnia than without (regardless of melancholic features): 90.1% vs. 19.4% respectively (*p* < 0.001).	Controlled group: depressed patients without melancholic features (*n* = 75)
Agargun, 2007 [32]	Controlled study	To examines the relationship among nightmares, suicide attempts and melancholic features in unipolar major depressed patients	MDD, *n* = 100	32.1	51% (*n* = 51)	DSM-IV criteria	ICSD-R criteria. All patients received an oral definition of nightmare Nightmare frequency Frequent nightmare if ≥ 2/week	Suicide attempt during current episode (≥1) HDRS (items 6, 7 and 8 for insomnia)	MDD with melancholic features: 28% (*n* = 28) suicide attempt during current episode; significant difference between melancholic patients with and without suicidal attempt in terms of frequency of middle and terminal insomnia and nightmares (nightmares, *n* = 27 (96%) in suicide attempt group, vs. *n* = 57 (79%) in non-suicide attempt group (*p* = 0.036)). No significant difference in the non-melancholic group for suicide attempt in term of nightmares and insomnia. Nightmares more common in MDD with melancholic feature (*n* = 84, 84%) than non-melancholic features (*n* = 28, 57%); *p* = 0.001. Nightmares more frequent in patients with suicidal attempt (*n* = 33.86%), than non-attempters (*n* = 79, 71%); *p* = 0.039.	Controlled group: depressed patients without melancholic features (*n* = 49)
Thünker, 2012 [33]	Cohort	To test the effectiveness of standardized nightmare therapy (based on IRT) in patients suffering from nightmares only, or with depression or PTSD.	MDD, *n* = 21	30.0	-	ND	Self-questionnaire (nightmare frequency, anxiety during nightmares and daytime distress (7-point scale))	Overall improvement of therapy on a 7-point scale	Decrease of nightmare frequency after intervention in MDD patients; decrease of anxiety after intervention	8 therapy session of 50 min each
Li, 2012 [34]	Prospective cohort	To investigate the prevalence and persistence of nocturnal sleep symptoms, and examine sociodemographic, clinical and psychosocial correlates of residual sleep disturbances in remitted depressed patients and investigate association of functional outcomes and suicidal ideation with residual sleep disturbances in remitted depressed patients.	362	44.6	81.7% (*n* = 296)	ICD-10 criteria	Sleep questionnaire for insomnia and nightmare (5-point scale) Nightmare Distress Questionnaire (NDQ)	Suicide evaluation: MINI suicidality module	Patients with nightmares and insomnia had fewer chance to be in remission of MDD at 4 years (29.8% of remission in patients with nightmares (*n*= 36) vs. 47.3% of remission in patients without nightmares (*n* = 114), *p* < 0.01). Frequent nightmare at baseline was significantly associated with residual nightmare at follow up (OR = 5.17, 95% CI (13.35–19.9)) after controlling for sociodemographic factors and psychiatric comorbidity. Suicidal ideation was significantly associated with residual nightmares (OR = 8.40, 95% CI (1.79–39.33)) after controlling for confounding factors	There were no definition of nightmares
Marinova, 2014 [22]	Cohort	To test the hypothesis that nightmares are associated with an elevated suicidal risk in depressed patients	RDD, *n* = 44 BD, *n* = 8	24 to 75	53.8% (*n* = 28)	ICD-10 criteria	Nightmare: defined as unpleasant dream Dream questionnaire Nightmare yes/non (no measure of frequency)	Question 3 of HDRS for suicidality	Difference between RDD and BD: 64% of RDD had nightmares vs. 25% of BD (*p* < 0.044). In RDD subgroup, patients with nightmares had significantly higher average score on the HDRS item on suicide risk (2.36) than those without nightmare (1.00), *p* < 0.005. They had history of suicide attempts significantly more frequently (35% vs. 6%, *p* < 0.05). No differences in the BD subgroup.	No differentiation bad dreams and nightmares
Woo, 2014 [35]	Case report	To describe EMDR treatment of nightmare and highlights various aspects of EMDR therapy	MDD, *n* = 1	36	100% (*n* = 1)	Clinical examination	4–5/weeks for 9 years	-	Better after four sessions of EMDR	-
Lai, 2014 [36]	Cohort	To examine whether patients with BD and MDD exhibit different sleep problems and to what extent their relatives have sleep complaints	BD, *n* = 363 MDD, *n* = 157	35.5 (BD), 45.6 (MDD)	52.3% (*n* = 190) for BD, 68.9% (*n* = 108) for MDD	DSM-IV criteria	PSQI for sleep quality	-	Frequent nightmares in patients with MDD or BD were associated to a higher risk of suicidal ideations (OR = 2.88) and attempts (OR = 1.89) after adjustment for age at interview and sex.	-

Table 1—Mood disorders and nightmares. F = Female; RDD = Recurrent Depressive Disorder; BD = Bipolar Disorder, MDD = Major Depressive Disorder; ME = Manic Episode; ND = No Data; PSQI = Pittsburgh Sleep Quality Index; DSM = Diagnostic and Statistical Manual of Mental Disorders; ICD-10 = International Statistical Classification of Diseases and Related Health Problems; SADS = Schedule for Affective Disorders and Schizophrenia; BDI: Beck Depression Inventory; HDRS: Hamilton Depression Rating Scale.

**Table 2:** Psychotic Disorders and nightmares.

Authors Years	Type of Article	Aims	*n*	Age (Years)	Sex F	Diagnosis Criteria	Nightmares: Diagnosis and Severity	Other Evaluation	Main Results	Comments
Shorkey, 1974 [37]	Case report	Treatment of recurrent nightmare with desensitization	Schizophrenia, *n* = 1	38	0% (*n* = 0)	Clinical examination	Daily nightmares	-	In the nightmare, the devil was a snake; treatment by desensitization of fears of snakes, with efficacy on nightmares (no nightmares avec 2 years follow up)	
Fennig, 1992 [38]	Brief Report	Presentation of a patient which had a transition from nightmare to brief psychotic episode	BPE, *n*= 1	78	0% (*n* = 0)	Clinical examination	Recurrent nightmare for 3 years	-	Brief psychotic episode occurring after a nightmare, with same thematic; treatment by TRIAZOLAM 25 mg/d and OXAZEPAM 20 mg/d with no recurrence after 6 months follow up	
Levin, 1998 [39]	Case report	To illustrate how the nightmare may presage the underlying psychological disorganization during the period of psychic decompensation	Schizophrenia, *n* = 1	40	100% (*n* = 1)	DSM-IV criteria		-	Insomnia and nightmare the week before relapse; same thematic of delusion and hallucination, than in nightmares preceding decompensation.	The patient remained able to differentiate her nightmares from her waking hallucinations and her waking life.
Lusignan, 2009 [40]	Controlled study	To investigate dream content in patients with schizophrenia using both questionnaire and laboratory REM sleep awakenings	Schizophrenia, *n* = 14	25.5	(*n* = 1)	DSM-IV-TR criteria	Oral definition of nightmares Dream questionnaire (11 items self-report): nightmare frequency and dream emotions	3 consecutive nights in sleep laboratory; dream report collected following awakenings from REM sleep	Self-report questionnaire: patients with schizophrenia reported nightmares more frequently (4.1 vs. 2.1, *p* = 0.006) and more nightmare per year (22.8 vs. 3.5, *p* = 0.003). No significant difference for bad dreams, dream recall, lucid dreaming, control of dreams or physical sensation during dreams. Laboratory measures: 92 awakenings from REM sleep, with 78 dream reports (no difference between 2 groups in dream reports, and white dream)	Control group, *n* = 15. No difference in polysomnographic measures in both groups.
Michels, 2014 [41]	Controlled study	To investigate nightmare frequency and its correlates in patients with schizophrenia	Schizophrenia, *n* = 17	32.9	47.0% (*n* = 8)	ND	DDNSI for frequency of nightmares (0 = never to 7 = several times a week) and intensity (distress)	ISI for insomnia C-SSRS for suicide BDI	Higher frequency of nightmares in patients with schizophrenia (3.65, *p* = 0.0145), and ARMS patients (3.79, *p* = 0.003) than HC No significant correlation between BDI and nightmare frequency No significant correlation between PANNS and nightmare frequency	Comparison with 14 patients with at risk mental states for psychosis (ARMS) and 17 healthy relatives of patients
Sheaves, 2015 [42]	Case series	To assess the acceptability and feasibility of Imagery Rehearsal (IR) for the treatment of nightmares in the context of psychosis	*p*, *n*= 6	39.7	66.6% (*n* = 4)	ND	7 points rating scale for nightmare intensity, vividness and distress, and if recurrent (yes/no) Mean of 4.4 per week	-	5/6 participants attended 4–6 sessions of IR. Mean nightmare related distress across the week decreased from 5.43 to 4.28; improvement on vividness and intensity of nightmares; no decrease of nightmare frequency, but patients described a change in emotional response.	Participants with PTSD were not excluded from the study IR method: psychoeducation, collaboratively planning a rescript of the nightmare, elaborating the rescript with sensory detail through guided imagery and daily practice of the new dream script; 4–6 sessions
Sheaves, 2015 [43]	Cohort	To examine the prevalence of nightmares in people with psychosis and to describe the link between nightmares and sleep quality, psychotic, affective and cognitive symptoms	*p*, *n*= 40	41.9	62.5% (*n* = 25)	ND	Nightmare frequency 7 points rating scale for nightmare intensity, vividness and distress, and if recurrent (yes/no)	PSQI for sleep quality	At least one nightmare in the past 14 nights in 28 patients (70%). 22/40 (55.0%) patients experienced at least weekly nightmares. Nightmare frequency was associated with poorer sleep: large positive correlation between nightmare frequency and PSQI while controlling for antipsychotic dose; nightmare frequency was negatively correlate with sleep efficiency. Nightmare distress, rather than frequency, is a best account for the association between nightmares and daytime impairment: correlation between nightmare distress and delusional severity, depression, anxiety, and stress.	*n* = 17 (42.5%) of patients screened positive for PTSD; 11/17 (65%) reported weekly nightmares. This was not significantly higher than the 10/20 (50%) participants who reported weekly nightmare but did not screen positive for PTSD. No difference in nightmare distress between PTSD and without PTSD.
Chiu, 2016 [44]	Cohort	To explore the lived experience of sleep problems in people with schizophrenia-spectrum disorders	14	ND	50% (*n* = 7)	ND		-	*n* = 7 (50%) of patients reported nightmares	-
Li, 2016 [45]	Cohort	To examine the prevalence of sleep disturbances, particularly frequent insomnia and nightmares, and their prospective associations with the risk of suicide attempts in patients with schizophrenia spectrum disorder	*p*, *n* = 388 (Schizophrenia *n* = 308)	41.0	54.9% (*n* = 213)	ICD-10 criteria	Frequent nightmare was defined as having nightmares of at least once a week in the past year Sleep questionnaire for insomnia and nightmares (5-point scale for frequency)	-	At baseline: 19.3% frequent insomnia and 9.0% frequent nightmares. Comorbid insomnia and nightmare reported in 3.4%. Patients with frequent nightmare were more likely to report frequent insomnia (37.1% vs. 17.6%, *p* < 0.01). Patients with frequent insomnia were more likely to have comorbid nightmare disturbances (17.3%, vs. 7.0%, *p* < 0.01). The complaint of frequent nightmares was associated with a lifetime history of suicide attempts (42.9% vs. 19.3%); it did not predict an increased risk of suicide attempt over the follow up period.	8 years observational study on consecutively recruited cohort of psychiatric outpatients with schizophrenia spectrum diagnosis Comorbidity of insomnia and nightmare disturbances was significantly associated with an increased risk of suicide attempts, not only in lifetime (*p* < 0.001), but also during the 8 years follow up period (*p* < 0.01).
Reeve, 2018 [46]	Cohort	To report on the presence, severity and treatment of sleep disorders in patients with non-affective psychosis	*p*, *n* = 60	23.7	35.0% (*n* = 21)	DSM-5	ICSD-2, ICSD-3 Diagnostic Interview for Sleep Patterns and Disorders	Actigraphy (wrist-based activity monitoring device) and sleep diary for 7 days	80% (*n* = 48) of participants received a positive screen or diagnosis for at least one sleep disorder: insomnia was the most frequent (*n* = 30. 50%), then nightmare (*n* = 29, 48.3%). Comorbidity: average of 3.3 sleep disorder per patient. *n* = 30 (33%) had bot insomnia and nightmares. Severity of nightmares: Mild *n* = 2 (6.9%), moderate *n* = 11 (37.9%), severe *n* = 16 (55.2%); Night terror, nightmare disorder and RLS were the disorders least commonly discussed with a clinician (50%).	No significantly difference in antipsychotic dose between patients with and without nightmares.
Sheave, 2019 [47]	Randomized Control trial	To test the potential benefits of imagery focused cognitive behavioral therapy (CBT) for nightmares on nightmare severity and persecutory delusions.	24 patients with weekly nightmares and persecutory delusions in the context of a diagnosis of non-affective psychosis.	41.0	42% (*n* = 10)		DDNSI Sleep Condition Indicator (SCI)	PSQI for sleep quality	Large effect size reductions in nightmares and insomnia post treatment (4 weeks) (DDNSI d = −1.06; SCI d = −1.4) maintained at follow up (8 weeks). Post-treatment improvements were observed in paranoia (GPTS), affective symptoms (DASS-21), dissociation (DES-B), and emotional wellbeing (WEMWBS). There were no changes in hallucinations (CAPS) or activity levels (time budget).	12 treatment, 12 control. Core technique was imagery rehearsal training. Additional strategies included: psychoeducation about nightmares, reducing pre-sleep hyperarousal, increasing coping skills, reducing preoccupation with nightmares, stabilizing REM sleep.

BPE = Brief Psychotic Episode; ND = No Data, *p* = Psychosis; PSQI = Pittsburgh Sleep Quality Index; ARMS: at risk mental states for psychosis; BDI: Beck Depression Inventory, DDNSI: Disturbing Dreams and Nightmare Severity Index; ISI: Insomnia Severity Index; C-SSRS: Columbia suicide rating scale.

**Table 3:** Studies comparing psychiatric disorders and nightmares.

Authors Years	Type of Article	Aims	Diagnosis	*n*	Age (Years)	Sex F	Diagnosis Criteria	Nightmares	Main Results
Sjöström, 2007 [48]	Cohort	To examine the prevalence of specific sleep disturbances in suicide attempters and to test the associations between specific sleep disturbances and suicidality	MDD, *n* = 55 (33.3%), other depression *n* = 20 (12.1%); psychotic disorder *n* = 11 (6.6%), alcohol/substance misuse disorder *n* = 41 (24.8%); anxiety disorder and other disturbance *n* = 27 (16.3%)	165	35.3	78% (*n* = 129)	DSM-IV	How often do you experience nightmare from 1 to 5 Uppsala Sleep Inventory (USI) for sleep disturbance	Frequent nightmares were less common in patients who scored 0 on all 5 suicidality items (12%) than in those scoring ≥1 on any suicidality item (45%); *p* <0.001
Sjöström, 2009 [19]	Prospective cohort	To determine whether those who reported sleep disturbances in general and frequent nightmares in particular were at increased risk of repeat suicide attempt	MDD, *n* = 55 (33.3%), other depression *n* = 20 (12.1%); psychotic disorder *n* = 11 (6.6%), alcohol/substance misuse disorder *n* = 41 (24.8%); anxiety disorder and other disturbance *n* = 27 (16.3%)	165	35.3	78% (*n* = 129)	DSM-IV	How often do you experience nightmare from 1 to 5	Repeaters had more frequent nightmare than non-repeaters (*n* = 22 (52%) vs. *n* = 26 (30%), *p* = 0.002). 98 patients completed a 2 month follow up: persistent nightmare were about 3 times more common in repeaters (*n* = 13 (46%), vs. *n* = 10 (14%) *p* = 0.001)
Mume, 2009 [25]	Controlled study	To highlight nightmare in healthy individuals and to demonstrate its possible association with psychopathology, using schizophrenia and depressive episode as examples	Schizophrenia (*n* = 54), major depressive disorder (*n* = 40)	94	42.6 (schizophrenic patients), 43.1 (MDD patients)	-	-	Did you experience nightmare in the past one year? Yes/No/ if yes, how many times did you experience it in the past one year?	9/54 (16.7%) of schizophrenic patients experienced nightmare in the previous years, and 7/40 (17.5%) MDD patients (vs 6/123 (4.9%) HC). Significantly more frequent in psychiatric patients (17%) than HC (*p* = 0.0007); no significant difference between schizophrenic and MDD patients. Number of nightmare schizophrenic patients mean 42.7 (sd = 6.3); 44.6 (sd = 5.9) for MDD, and 18 (sd = 6.6) for HC, *p* < 0.05.
Lamis, 2018 [49]	Cohort	To test the hopelessness theory of suicide risk in psychiatric patients who may or may not experiencing nightmares.	Non-affective psychosis (23.3%), Bipolar I (26.2%), bipolar II (3.5%), MDD (11.6%), schizoaffective disorder (23.3%), other (12.8%)	172	39.15	*n* = 91	DSM-IV-TR criteria + MINI Beck Hopelessness Scale (BHS)	DDNSI for frequency and severity of nightmares.	Patients who reported monthly to weekly nightmares (compared to patients who reported yearly or no nightmare) were younger and more likely to have been hospitalized for a recent suicide attempt. They also had higher scores on BHS and MINI suicide risk. Severe hopelessness in 44% of frequent nightmares vs. 22.6%

F = Female, MDD = Major Depressive Disorder; ND: no data.

**Table 4:** Synthesis of the findings from the literature.

			Main Results Regarding Nightmares	Studies
Mood disorders	Depression	Frequency	• Nightmares are more frequent in patients with MDD and melancholic features than without.	Besiroglu, 2004 [31]
				Agargun 2007 [32]
			• Nightmares are more frequent in unipolar depression than bipolar depression	Marinova, 2014 [22]
		Suicide	• Higher suicidality score in MDD patients with frequent nightmares * than without	Agargun, 1998 [29]
			• More suicidal ideation in MDD patients with frequent nightmares ** than without	Li, 2012 [34]
			• More suicidal attempts in MDD patients with melancholic features with frequent nightmares * than without	Agargun 2007 [32]
			• More suicidal ideation and attempts in unipolar MDD and bipolar MDD in patients with frequent nightmares ****	Lai, 2014 [36]
				Marinova, 2014 [22]
		Treatment	• Decrease of nightmare frequency and anxiety after IRT intervention in MDD patients	Thünker, 2012 [33]
			• Improvement of nightmare frequency inn 36 MDD patients after four sessions of EMDR	Woo, 2014 [35]
		Evolution	• Patients with frequent nightmares ** were less in remission at four years than those without nightmares	Li, 2012 [34]
	Mania		• Three patients had nightmares as prodromal symptoms of their first manic episode	Agargun, 2003 [30]
Psychotic disorders		Frequency	• Nightmares are more frequent in patient with schizophrenia than in healthy controls	Lusignan, 2009 [40]
				Michels, 2014 [41]
			• Frequency of frequent nightmares ** ranging from 9 to 55%	Sheaves, 2015 [43]
				Chiu, 2016 [44]
				Li, 2016 [45]
			• Comorbid insomnia and nightmares are frequent in schizophrenia ∘ Patients with frequent nightmares ** were more likely to report frequentinsomnia ∘ Patients with frequent insomnia were more likely to have comorbid nightmares	Li, 2016 [45]
			• Nightmares are the second most frequent sleep disorder, after insomnia, in patients with schizophrenia.	Reeve, 2018 [46]
		Symptoms	• Same themes of delusion and hallucination than in nightmares preceding psychotic decompensation (two reports)	Levin, 1998 [39]
				Fennig, 1992 [38]
			• No significant correlations between depressive symptoms (assessed with BDI) and nightmare frequency • No significant correlations between psychotic symptoms (assessed with PANNS) and nightmare frequency	Michels, 2014 [41]
			• Nightmare distress, rather than frequency, is the best marker for the association between nightmares and daytime impairment: significant correlations between nightmare distress and delusional severity, depression, anxiety, and stress.	Sheaves, 2015 [43]
		Suicide	• Patients with frequent nightmares ** have more lifetime of suicide history and increase risk of suicide attempts	Li, 2016 [45]
		Treatment	• Efficacy of treatment by desensitization (one report) • Improvement of IRT on emotional response, not on nightmare frequency	Shorkey, 1974 [37]
				Sheaves, 2015 [42]
			• Imagery focused cognitive behavioral therapy: large effect size reductions in nightmares and insomnia post treatment (4 weeks)	Sheave, 2019 [47]
Comparing studies		Frequency	• Significantly more frequent in psychiatric patients (schizophrenia and MDD) than healthy controls • No significant differences between patients with schizophrenia and MDD	Mume, 2009 [25]
		Suicide	• Higher suicidality scores (assessed with SUAS) in patients with frequent nightmares than without (MDD, schizophrenia)	Sjöström, 2007 [48]
			• Patients with more than one past suicide attempts had more frequent nightmares**** than those with first suicide attempts	Sjöström, 2009 [19]
			• Patients who reported monthly to weekly nightmares were younger and more likely to have been hospitalized for a recent suicide attempt. • Severe hopelessness more frequent in patients with nightmares	Lamis, 2018 [49]

SCZ: schizophrenia; MDD: Major Depressive Disorder; ME: Manic Episode; BDI: Beck Depression Inventory; PANNS: Positive and Negative Syndrome Scale; IRT: Imagery Rehearsal therapy. Table 4: Summary table of main results from the systematic review of nightmares in mood and psychotic disorders *MDD:* Major Depressive Disorder; BDI: Beck Depression Inventory; PANNS: Positive and Negative Syndrome Scale; IRT: Imagery Rehearsal therapy; EMDR: Eye Movement desensitization and reprocessing; SUAS: The Suicide Assessment Scale. Frequent nightmares were defined in some studies as: ≥2/week*, ≥1/week **. In the other studies, frequent nightmare was not defined ****.
